# Nicotinamide and Sugar Metabolism Associated With Muscle Mass Loss During Calorie Restriction in Older Adults

**DOI:** 10.1093/geroni/igaa057.415

**Published:** 2020-12-16

**Authors:** Ellen Quillen, Jingyun Lee, Cristina Furdui, Daniel Beavers, Kristen Beavers

**Affiliations:** 1 Wake Forest School of Medicine, Winston-Salem, North Carolina, United States; 2 Wake Forest Baptist Health, Winston-Salem, North Carolina, United States; 3 Wake Forest University, Winston-Salem, North Carolina, United States

## Abstract

Weight loss among older adults remains controversial due to lean mass loss and potential exacerbation of disability risk. Using the Medifast for Seniors clinical trial (NCT02730988), which investigated high protein supplementation (≥1.0 g/kg/d) during caloric restriction to preserve lean mass among 96 older adults (>70 years, 74% women, 27% black) with obesity (BMI: 35 kg/m^2), we applied untargeted metabolomics to identify small molecules associated with the highly variable change in lean muscle mass during weight loss. Forty-seven participants were randomized to high protein weight loss, and 92% lost at least 5% body weight over 24 weeks. Across DXA-ascertained measures of lean body mass, gynoid lean mass exhibited the broadest range of change: +4% to -12%. For 38 participants, untargeted metabolomics data was generated from fasted serum samples collected before and after intervention. 121 serum metabolites were identified and change from baseline was tested for correlation with percent change in gynoid muscle mass. Increasing nicotinamide levels were associated with a greater loss of gynoid muscle mass (R^2=0.22, p=0.0027). Pathway analysis was applied to identify Kyoto Encyclopedia of Genes and Genomes (KEGG) biochemical pathways containing multiple nominally-associated metabolites. The amino sugar and nucleotide sugar metabolism pathway was significantly enriched (p=0.006), containing four sugar metabolites associated with shifts in lean muscle mass. This pathway is important in the glycosylation of polysaccharides, a ubiquitous and important regulator of energy metabolism, but has not previously been linked to muscle mass and should be further interrogated in preservation of lean muscle during weight loss.

